# EPIDEMIOLOGICAL INVESTIGATION OF TRAUMATIC SPINAL CORD INJURY CAUSED BY OBJECT STRIKE IN CHINA: STRATEGIES FOR WORKPLACE SAFETY IMPROVEMENT

**DOI:** 10.2340/jrm.v56.40880

**Published:** 2024-11-12

**Authors:** Wenjie ZHANG, Fangyong WANG, Zezheng CHEN, Yang YU, Tao LIU, Honghui LEI, Haoran YIN, Meiling CHENG

**Affiliations:** 1 School of Rehabilitation Medicine, Capital Medical University, Beijing; 2 China Rehabilitation Research Center, Beijing; 3 University of Health and Rehabilitation Sciences, Qingdao, Shandong; 4 Wenzhou Medical University, Wenzhou, Zhejiang

**Keywords:** complications, epidemiology, occupations, safety, spinal cord injury, workplace

## Abstract

**Objective:**

Being struck by an object is a major cause of traumatic spinal cord injury in China. This study aims to investigate epidemiological characteristics of spinal cord injury caused by object strike.

**Methods:**

This research analysed data from 435 cases of strike-induced spinal cord injury from 2013 to 2022. The collected information encompassed gender, age, level of neurological injury, surgical interventions, expense, occupation, and other relevant factors. χ^2^tests and Mann–Whitney *U* test were used with a statistical significance level of 0.05.

**Results:**

The male-to-female ratio was 11.8:1. The 30–44 age group was more likely to suffer from complete spinal cord injuries (70.5%). The predominant occupations were workers (58.9%) and farmers (15.2%). Manual labourers are usually injured in the workplace (89.4%) with a high surgical rate (95.3%).

**Conclusion:**

Young and middle-aged males engaged in manual work constitute the primary demographic for strike-induced spinal cord injury. Safety education in workplaces such as construction sites and mines should be emphasized to reduce the occurrence of spinal cord injuries caused by object strikes.

## LAY ABSTRACT

This study aims to investigate epidemiological characteristics of spinal cord injury caused by object strike. This research analysed data from 435 cases of strike-induced spinal cord injury from 2013 to 2022 and collected information encompassing gender, age, level of neurological injury, surgical interventions, expense, occupation, and other relevant factors. The male-to-female ratio was 11.8:1. The 30–44 age group was more likely to suffer from complete spinal cord injuries (70.5%). Workers (58.9%) and farmers (15.2%) were the predominant occupations. Manual labourers are usually injured in the workplace (89.4%) with a high surgical rate (95.3%). Young and middle-aged males engaged in manual work constitute the primary demographic for strike-induced spinal cord injury. Safety education in workplaces such as construction sites and mines should be emphasized to reduce the occurrence of spinal cord injuries caused by object strikes.

Traumatic spinal cord injury (TSCI) constitutes a profoundly debilitating condition characterized by an abrupt, multi-system disorder that results in the irreversible disruption of homeostasis and neurological impairments beneath the injury site, culminating in substantial functional loss and potential permanent disability (1–3). Between 2000 and 2021, the global incidence of TSCI remained alarmingly high, ranging from 20 to 45 cases per million individuals (4). Asystematic review revealed that the pooled incidence of TSCI in China was estimated at 65.15 per million (5). The aetiological factors contributing to TSCI included falls from height (37.5%), traffic accidents (26.9%), being struck by an object (16.3%), and falls on the ground (8.3%) (6). Object strikes rank among the primary causes of TSCI in China.

The incidence of spinal cord injuries attributable to object strikes exhibited regional variability across China, yet it revealed distinct occupational trends. The proportion of spinal cord injuries resulting from such impacts was notably higher in Guangdong (19.5% from 2003 to 2011), Beijing (18.6% in 2002), and Chongqing (13.2% from 2009 to 2013) compared with other areas (7). A hospital-based investigation into the epidemiological characteristics of spinal cord injuries in northwestern China indicated that the occupations most vulnerable to these injuries were those of peasants and workers. Among a cohort of 3,487 patients with spinal cord injuries, peasants and workers constituted a significant 59.51% and 27.04%, respectively (8). Furthermore, a study focusing on the epidemiological profile of TSCI at Tianjin Medical University General Hospital corroborated these findings, revealing that peasants, workers, and unemployed individuals faced elevated risks of TSCI, with occupations of peasants and labourers representing 32.5% and 24.3%, respectively (9). The ongoing economic development has consequently led to an increase in the proportion of manual labourers, further intensifying these risks.

Although study of TSCI caused by falling or traffic accidents is easily available, investigation of epidemiological characteristics of spinal cord injury caused by object strike is rare. This study aims to investigate epidemiological characteristics of TSCI caused by object strike and to propose corresponding preventive measures.

## METHODS

### Study design

This retrospective study was conducted at the China Rehabilitation Research Center, the nation’s premier rehabilitation institution, which admits thousands of patients requiring rehabilitation from across China each year. The study focused on a cohort of 435 patients with TSCI caused by object strikes, admitted between 1 December 2012, and 30 November 2022. Their medical records were meticulously reviewed and independently evaluated by 2 researchers to ensure a comprehensive and unbiased analysis.

### Processes

Two independent researchers examined the consultation records and imaging examinations to identify cases of TSCI caused by object strike. Patient details, including age, gender, occupation, neurological level, and severity of injury at admission, complications, and rehabilitation length of stay (LOS) were obtained from the medical record system.

There were 401 male cases and 34 female cases with a mean age of 39.2 years. They were categorized into 4 age groups: 15–29 years, 30–44 years, 45–59 years, and ≥60 years, to examine the characteristics of TSCI caused by object strike across different age demographics. The severity of the injuries was documented using the American Spinal Injury Association (ASIA) classification, categorizing them into complete injuries (Grade A) and incomplete injuries (Grades B, C, and D). Participants engaged in manual labour, including workers and farmers, were grouped into the manual work category, while those in professions such as civil service, education, and corporate employment were classified as non-manual workers to analyse occupational characteristics. Complications associated with TSCI encompassed urinary infections, neurogenic bladder, intestinal dysfunction, neuralgia, spasticity, osteoporosis, pressure ulcers, respiratory infections, and deep vein thrombosis. The epidemiological characteristics of TSCI caused by object strikes were evaluated by synthesizing data on age, gender, injury severity, neurological level, spinal surgery, complications, and overall costs.

Ethical permission was obtained at the authors’ institution. Informed consent was waived by the ethical review boards as this is a retrospective study.

### Statistical analysis

Data analysis was performed using SPSS software (version 25.0; IBM Corp, Armonk, NY, USA) to assess relevant variables. Demographic factors, including gender, age, injury location, and payment method, as well as clinical variables such as injury severity, extent of injury, affected segments, and surgical treatment, were classified as categorical variables, while LOS was regarded as a continuous variable. Categorical variables were analysed using the χ test, whereas differences in LOS between the2 groups were evaluated using the Mann–Whitney *U* test, with a significance level set at 0.05. Descriptive statistics, including median and interquartile range (IQR), frequency, and percentage, were employed to describe the demographic and clinical characteristics of the study subjects who sustained injuries resulting from object strikes.

## RESULTS

### Characteristics of different age range groups

This study included 401 male and 34 female participants, yielding a mean age of 39.2 years (11.2). The male-to-female ratio was 11.8: 1. Table I illustrates the epidemiological characteristics across different age groups, with a peak age distribution observed in the 30–44 years bracket, accounting for 43.7%, followed by the 45–59 years group (29.4%). The incidence of paraplegia was higher than that of quadriplegia across all demographics. Notably, the youth group (15–29 years) exhibited the highest incidence of paraplegia (86.0%), while the elderly group demonstrated the lowest rate (58.8%). Individuals in the 15–29 years cohort were significantly more likely to experience ASIA A spinal cord injuries (75.0%), while the likelihood of ASIA D injuries was considerably lower (7.0%). The overall surgical rate was notably high at 91.5%, with both the 30–44 years group (94.7%) and the 15–29 years group (92.0%) exceeding 90%. Furthermore, the likelihood of choosing out-of-pocket payment for hospital expenses among those aged over 60 (41.2%) was significantly lower than that of the 30–44 years group (64.7%).

**Table I:** Characteristics of 435 cases according to different age range groups

Characteristics	15–29 years	30–44 years	45–59 years	≥ 60 years	Total	*p*-value
Gender						χ^2^ = 1.638, *p* = 0.651
Male (401)	91 (91.0)	174 (91.6)	121 (94.5)	15 (88.2)	401 (92.2)	
Female (34)	9 (9.0)	16 (8.4)	7 (5.5)	2 (11.8)	34 (7.8)	
Neurological level of injury						χ^2^ = 25.532, *p* < 0.001
Tetraplegia	14 (14.0)	37 (19.5)	50 (39.1)	7 (41.2)	108 (24.8)	
Paraplegia	86 (86.0)	153 (80.5)	78 (60.9)	10 (58.8)	327 (75.2)	
ASIA						χ^2^ = 9.993, *p* = 0.019#
A	75 (75.0)	134 (70.5)	75 (58.1)	9 (52.9)	293 (67.2)	
B	5 (5.0)	15 (7.9)	16 (12.4)	1 (5.9)	37 (8.5)	
C	13 (13.0)	23 (12.1)	21 (16.3)	3 (17.6)	60 (13.8)	
D	7 (7.0)	18 (9.5)	17 (13.2)	4 (23.5)	46 (10.6)	
Complication						χ^2^ = 7.444, *p* = 0.057
Yes	80 (84.1)	165 (86.8)	110 (85.9)	11 (64.7)	366 (84.1)	
No	20 (15.9)	25 (13.2)	18 (14.1)	6 (35.3)	69 (15.9)	
Spine surgery						χ^2^ = 9.228, *p* = 0.029
Yes	92 (92.0)	180 (94.7)	113 (88.3)	13 (76.5)	398 (91.5)	
No	8 (8.0)	10 (5.3)	15 (11.7)	4 (23.5)	37 (8.5)	
Length of stay (days)						χ^2^ = 3.964, *p* = 0.265
3 months	46 (46.0)	107 (56.3)	80 (58.0)	10 (58.8)	243 (54.6)	
≥ 3 months	54 (54.0)	83 (43.7)	58 (42.0)	7 (41.2)	202 (45.4)	
Payment method						χ^2^ = 8.367, *p* = 0.039
Own expense	50 (50.0)	123 (64.7)	78 (60.9)	7 (41.2)	258 (59.3)	
Other	50 (50.0)	67 (35.3)	50 (39.1)	10 (58.8)	177 (40.7)	
Total cost (million)						χ^2^ = 1.699, *p* = 0.637
0–0.1	44 (44.0)	98 (51.6)	65 (50.8)	9 (52.9)	216 (49.7)	
≥ 0.1	56 (56.0)	92 (48.4)	63 (49.2)	8 (47.1)	219 (50.3)	

#Kruskal–Wallis test.

### Distribution of neurological level of injury

**Fig. 1:**
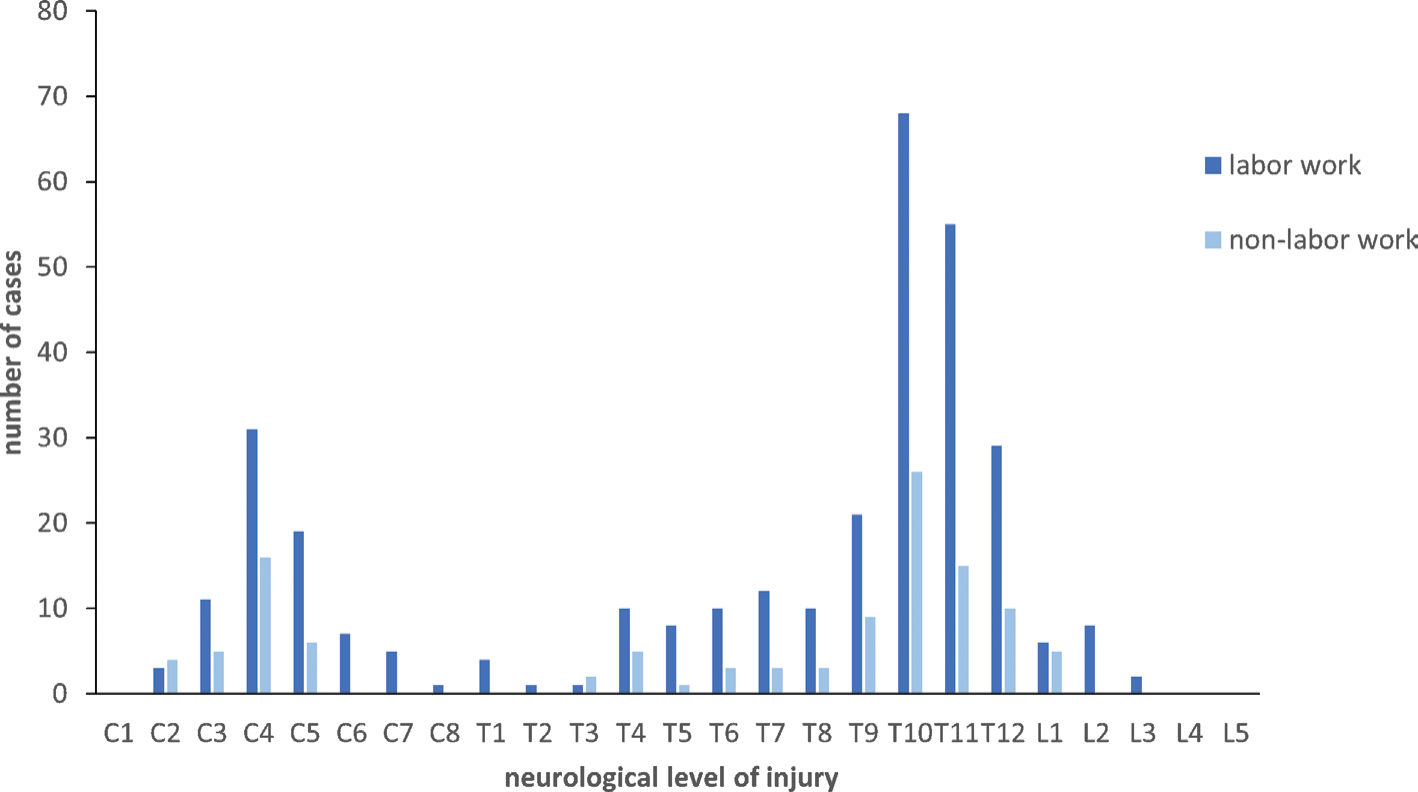
Number of cases with different neurological level of injury.

As shown in Fig. 1, the distribution of neurological level of injury (NLI) in cases of TSCI predominantly centred on the thoracic region, with T10 (21.6%) and T11 (16.1%) being the most affected. The most common cervical spinal cord injury was observed at C4, accounting for 10.8% of all cases. Lumbar spinal cord injuries were the least frequent. In the manual labour group, the NLI was primarily concentrated in T10 (21.1%) and T11 (17.1%), with a smaller peak at C4 (14.3%). In contrast, in the non-labourer group, the most common NLI was T10 (26.5%), followed by C4 (14.1%).

### Occupations

**Fig. 2:**
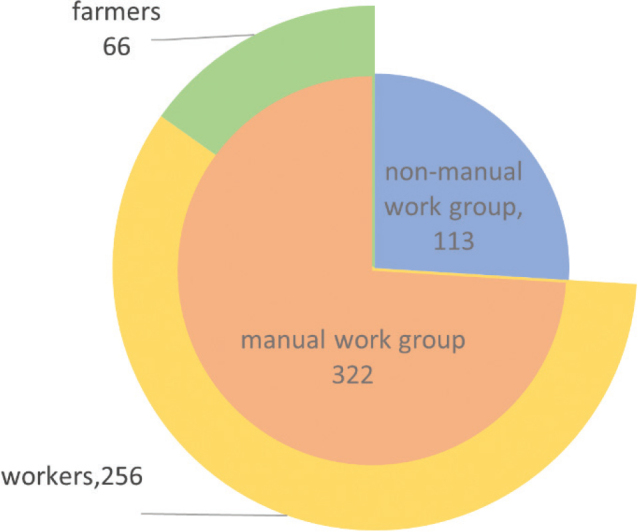
Number of cases with different occupations.

Fig. 2 illustrates the distribution of cases across different occupations. The predominant professions were labourers (58.9%) and farmers (15.2%). Themanual labour group comprised 322 cases, while the non-manual labour group included 113 cases. Table II reveals statistically significant differences between manual and non-manual labourers in terms of age range, gender, complications, payment method, spinal surgery, and work-related injuries. The age distribution in the manual labour group (mean 39.44, SD 10.50) was concentrated in the 30–44 and 45–59 age brackets, accounting for 76.7%. Compared with manual labourers, the proportion of individuals aged over 60 was higher in the non-manual labour group. Both groups had a higher proportion of males. However, the gender ratio in the manual labour group (22.3) was significantly higher than in the non-manual labour group (4.7) (χ² = 20.694, *p *< 0.001).

**Table II:** Characteristics of 435 participants engaging in manual work and non-manual work

Characteristics	Manual-work group			Non-manual workgroup	*p*-value
Age					χ^2^ = 13.792, *p* = 0.003
15–29 years	68 (21.1)			32 (28.3)	
30–44 years	142 (44.1)			49 (43.4)	
45–59 years	105 (32.6)			23 (20.4)	
≥ 60 years	7 (2.2)			9 (8.0)	
Gender					χ^2^ = 20.694, *p* < 0.001
Male	308 (95.7)			93 (82.3)	
Female	14 (4.3)			20 (17.7)	
Neurological level ofinjury					χ^2^ = 0.555, *p* = 0.456
Tetraplegia	77 (23.9)			31 (27.4)	
Paraplegia	245 (76.1)			82 (72.6)	
ASIA					χ^2^ = 0.889, *p* = 0.374
A	212 (65.8)			80 (70.8)	
B	28 (8.7)			9 (8.0)	
C	48 (14.9)			12 (10.6)	
D	34 (10.6)			12 (10.6)	
Complications					χ^2^ = 2.389, *p* = 0.017
0–1	92 (28.6)			43 (38.1)	
2–3	121 (37.6)			45 (39.8)	
4–5	83 (25.8)			18 (15.9)	
≥ 6	26 (8.1)			7 (6.2)	
Spine surgery					χ^2^ = 23.577, *p* < 0.001
Yes	307 (95.3)			91 (80.5)	
No	15 (4.7)			22 (19.5)	
Payment method					χ^2^ = 6.534, *p* = 0.038
Own expense	194 (60.2)			64 (56.6)	
Work injury insurance	83 (25.8)			22 (19.5)	
Other	45 (14.0)			27 (23.9)	
Workplace injury					χ^2^ = 118.054, *p* < 0.001
Yes	288 (89.4)			44 (38.9)	
No	34 (10.6)			69 (61.1)	
Total cost (RMB, million)					χ^2^ = 3.561, *p* = 0.059
0–0.1	152 (47.2)			65 (57.5)	
≥ 0.1	170 (52.8)			48 (42.5)	
Length of stay (days)	88.0 (58.8, 115.3)			71.0 (38.0,108.5)	*p* < 0.05*

* Mann–Whitney *U* test.

Regarding payment methods, a greater proportion of manual labourers paid out-of-pocket (60.2%) compared with non-manual labourers (56.6%). Both groups demonstrated relatively low participation in work injury insurance, though the enrolment rate was marginally higher among manual labourers (25.8%) than their non-manual counterparts (19.5%) (χ² = 6.534, *p* < 0.05). Work-related injuries were notably more prevalent in the manual labour group, accounting for 89.4% of cases, whereas only 38.9% of non-manual labourers experienced work-related injuries. In terms of complications, a higher proportion of manual labourers experienced 4–7 complications (33.9%) compared with non-manual labourers (22.1%) (χ² = 2.389, *p* = 0.017). Furthermore, the incidence of spinal surgery was significantly greater among manual labourers (95.3%) relative to non-manual labourers (80.5%) (χ² = 23.577, *p* < 0.001). These findings collectively suggested that manual labourers tended to have longer hospital stays overall.

### Complications

Table III presents the incidence of complications associated with TSCI in this study. Neurogenic bladder was the most common complication, with higher rates observed in the 30–44 age group (64.4%) and the 45–59 age group (56.6%). Urinary tract infections were more prevalent in the 15–29 age group (64.4%) and those aged over 60 (56.6%). Neuropathic pain, spasticity, and intestinal dysfunction were also frequent complications among patients with TSCI resulting from object strikes.

**Table III:** Incidence of complications among patients of different ages

Complication	15–29 years	30–44 years	45–59 years	≥ 60 years
Urinary infection	50 (50.0)	81 (44.3)	30 (35.7)	10 (58.8)
Neurogenic bladder	43 (43.0)	122 (64.4)	47 (56.0)	9 (52.9)
Intestinal dysfunction	25 (25.0)	72 (38.4)	23 (27.4)	4 (23.5)
Neuralgia	36 (36.0)	94 (49.5)	44 (52.4)	8 (47.1)
Spasticity	29 (29.0)	82 (43.2)	12 (14.3)	4 (23.5)
Osteoporosis	27 (27.0)	63 (33.2)	17 (20.2)	4 (23.5)
Pressure ulcer	8 (8.0)	42 (22.1)	11 (13.1)	4 (23.5)
Respiratory infection	4 (4.0)	14 (7.4)	8 (9.5)	4 (23.5)
Deep vein thrombosis	3 (3.0)	20 (10.5)	9 (10.7)	1 (5.9)

## DISCUSSION

Being struck by object is one of the main causes of TSCI in China. Considering China’s huge population, the number of cases with TSCI caused by object strike cannot be ignored.

Research indicated that TSCI was more prevalent in men than in women; a systematic review revealed that the incidence of TSCI in males was 3.2 times that in females, corroborating the observed gender ratio of approximately 3:1 among TSCI patients (4). We found that in this survey the male-to-female ratio (11.8) was much higher than the ratio of TSCI caused by traffic accidents (2.7) and falls (4.6) (10, 11). This elevated sex ratio could be attributed to various factors. Menwere more likely to be employed in industries with heightened occupational hazards, such as construction, manufacturing, and transportation. These occupations exposed workers to an increased risk of falls, heavy machinery accidents, and other work-related incidents that may lead to spinal cord injuries (12). This pattern was further validated by the occupational characteristics of TSCI patients in our study, who predominantly worked as labourers and farmers (74%), with a male-to-female ratio reaching 22:1. Additionally, developing countries exhibited higher male-to-female ratios in TSCI cases (13). Based on global data, the age distribution of patients with traumatic SCI was bimodal; the first peak was between 15 and 29 years old, and the second smaller but growing peak was in those over 50 years old (14). With economic development and an ageing population, TSCI showed an ageing trend (15). Despite the ageing problem in China, the patients showed a tendency to be younger (39.2) in the investigation of TSCI caused by object strikes.

Globally, cervical spinal cord injuries accounted for more than 50% of TSCI, with their incidence significantly surpassing that of thoracic and lumbar injuries (16). Epidemiological research on TSCI in China reported that most injuries occurred in the cervical spine (61.3%), with incomplete quadriplegia being the most frequent presentation (44.2%) (17). However, in our survey, we observed that the thoracic spine was the most common site of injury, and younger patients were more likely to suffer from complete paraplegia. This finding aligned with an Australian study on workers with TSCI, which reported paraplegia in 68% of cases and tetraplegia in 32% (18). The level and severity of spinal cord injuries were crucial predictors of clinical outcomes. Previous studies have indicated that injuries at lower spinal levels tend to result in better prognoses (19). For instance, patients classified as Grade A on the American Spinal Injury Association (AIS) scale, which denotes complete injury, had only an 8.3% likelihood of walking independently 1 year post-injury. Conversely, those with Grade D injuries, indicating partial function, had a much higher probability (97.3%) of regaining independent mobility (20). These findings underscore the significant challenges faced by young patients with complete SCI, who are unlikely to regain independent ambulation.

Previous research has indicated that the proportion of patients with TSCI undergoing surgical intervention varied between 36.4% and 59.1% across different WHO regions (21). Surgical treatment appeared to be more prevalent among older patients (22). Our study revealed a surgery rate of 76.5% for those aged 60 and above, and an even higher rate of 94.7% among individuals aged 30–44. The primary factors guiding surgical intervention included spinal compression and neurological deficits (23). We speculated that the higher surgery rate may result from the CRRC accepting patients with severe spinal cord injuries nationwide, where significant spinal compression and neurological dysfunction were prevalent.

Surgery often led to extended hospitalization (24). In comparison with TSCI resulting from falls and traffic accidents, patients injured by object strikes exhibited longer LOS (10, 11). Workers who were labourers also experienced a longer LOS than those who were non-labourers. The severity of the SCI markedly influenced LOS, with more severe injuries correlating with prolonged hospitalization (25). Furthermore, LOS was significantly associated with complications such as neurogenic bladder, intestinal dysfunction, urinary tract infections, neuropathic pain, and respiratory infections, all of which were known to extend recovery time (26). The prevention, early diagnosis, and treatment of secondary complications in patients with TSCI were pivotal in mitigating the occurrence of these conditions, enhancing patient survival, reducing hospital stays, and improving health-related quality of life (27). In our survey, the most common complications of TSCI were neurogenic bladder and urinary tract infections. Urological issues were among the leading causes of re-hospitalization for individuals with TSCI, highlighting the critical importance of bladder management for these patients. Accurate diagnosis through urodynamic studies was essential for effective treatment and management (28, 29).

Given that labourers constituted over 70% of this study’s participants, and nearly 90% of these workers sustained injuries in the workplace, the implementation of targeted preventive measures for occupational spinal cord injuries was imperative. Ensuring occupational injury protection was essential to safeguard the fundamental rights of workers (30). The coverage rate of work-related injury insurance for labourers exposed to high occupational risks must be improved. Chinese migrant workers exhibited low awareness of health risks, with their work-related injury insurance coverage at just 23.6% (31). A survey conducted in the United States indicated that patients with traumatic spinal fractures were more likely to receive surgical treatment if they had insurance coverage (32). Based on this, we would advocate for the mandatory implementation of work-related injury insurance for migrant workers, along with encouraging individual farmers to purchase social insurance.

Primary prevention of work-related spinal cord injuries should be given substantially greater emphasis (33). A study on coalminers in Shanxi revealed that object strikes were the most common type of injury, comprising 53.63% of all incidents. Several risk factors for non-fatal occupational injuries included male gender, age, heavy manual labour, and working on underground front lines (34). Given these risks, targeted safety education was essential, particularly for labourers, with a specific focus on young and middle-aged men. Young workers (aged 15–24 years) experienced higher rates of work-related injuries compared with those aged 25–44. This increased risk may be attributed to limited or non-existent prior work experience or safety training. Therefore, providing adequate safety training and education for younger workers is crucial in reducing occupational injury rates and ensuring safer work environments (35).

The restoration of impaired neurological function and structural integrity following SCI remains an enduring challenge (36). Given that the majority of patients in this study sustained complete SCI, effective rehabilitation during the later stages is of paramount importance. Manella et al. have documented a case report that highlights significant improvements in ground walking ability after intensive physical therapy and robotic-assisted gait training (37). Additionally, another study found that exoskeleton-assisted walking activates the pelvic floor muscles, underscoring the therapeutic potential of such technologies (38). While over 95% of SCI patients at CRRC have undergone physical therapy, further advancement and integration of programs like robotic movement training are essential to optimize rehabilitation outcomes.

In terms of limitations in this study, first, the sample is concentrated in 1 hospital and cannot represent all patients with spinal cord injury. Also, some patients’ information was lost and screened out. Second, patient information such as occupation and place of injury was recorded manually by researchers and may be inaccurate. Third, misclassification of AIS at the time of the initial examination or follow-up may lead to change from neurologically complete to incomplete status. Lastly, as to the method’s limitation, confounders are likely to be part of our results considering the choice of methods itself.

In summary, the incidence of spinal cord injury resulting from object strikes predominantly affects young and middle-aged men engaged in physically demanding labour. Among all affected populations, complete paraplegia is the primary outcome, with high rates of complications, surgical interventions, and extended hospital stays. Thus, it is crucial to expand the coverage of work-related injury insurance and to enhance safety education in high-risk environments such as construction sites and mines. Moreover, improvements in rehabilitation treatment measures are vital to optimize recovery outcomes for these individuals.
